# Scalable and shapable nacre-like ceramic-metal composites based on deformable microspheres

**DOI:** 10.1093/nsr/nwaf006

**Published:** 2025-01-20

**Authors:** Yu-Jie Lu, Xiang-Sen Meng, Qiu-An Sun, Jie Wang, Jun-Jie Song, Peng-Fei Wang, Guo-Rui Wang, Cheng-Xin Yu, Yong-Sheng Zhang, Li-Bo Mao, Shu-Hong Yu

**Affiliations:** Department of Chemistry, New Cornerstone Science Laboratory, Institute of Biomimetic Materials & Chemistry, Anhui Engineering Laboratory of Biomimetic Materials, Division of Nanomaterials & Chemistry, Hefei National Research Center for Physical Sciences at the Microscale, University of Science and Technology of China, Hefei 230026, China; Department of Chemistry, New Cornerstone Science Laboratory, Institute of Biomimetic Materials & Chemistry, Anhui Engineering Laboratory of Biomimetic Materials, Division of Nanomaterials & Chemistry, Hefei National Research Center for Physical Sciences at the Microscale, University of Science and Technology of China, Hefei 230026, China; State Key Laboratory of Solid Lubrication, Lanzhou Institute of Chemical Physics, Chinese Academy of Sciences, Lanzhou 730000, China; Department of Chemistry, New Cornerstone Science Laboratory, Institute of Biomimetic Materials & Chemistry, Anhui Engineering Laboratory of Biomimetic Materials, Division of Nanomaterials & Chemistry, Hefei National Research Center for Physical Sciences at the Microscale, University of Science and Technology of China, Hefei 230026, China; State Key Laboratory of Solid Lubrication, Lanzhou Institute of Chemical Physics, Chinese Academy of Sciences, Lanzhou 730000, China; Department of Modern Mechanics, CAS Key Laboratory of Mechanical Behavior and Design of Materials, University of Science and Technology of China, Hefei 230026, China; Department of Modern Mechanics, CAS Key Laboratory of Mechanical Behavior and Design of Materials, University of Science and Technology of China, Hefei 230026, China; State Key Laboratory of Nonlinear Mechanics, Institute of Mechanics, Chinese Academy of Sciences, Beijing 100190, China; Department of Chemistry, New Cornerstone Science Laboratory, Institute of Biomimetic Materials & Chemistry, Anhui Engineering Laboratory of Biomimetic Materials, Division of Nanomaterials & Chemistry, Hefei National Research Center for Physical Sciences at the Microscale, University of Science and Technology of China, Hefei 230026, China; State Key Laboratory of Solid Lubrication, Lanzhou Institute of Chemical Physics, Chinese Academy of Sciences, Lanzhou 730000, China; Department of Chemistry, New Cornerstone Science Laboratory, Institute of Biomimetic Materials & Chemistry, Anhui Engineering Laboratory of Biomimetic Materials, Division of Nanomaterials & Chemistry, Hefei National Research Center for Physical Sciences at the Microscale, University of Science and Technology of China, Hefei 230026, China; Department of Chemistry, New Cornerstone Science Laboratory, Institute of Biomimetic Materials & Chemistry, Anhui Engineering Laboratory of Biomimetic Materials, Division of Nanomaterials & Chemistry, Hefei National Research Center for Physical Sciences at the Microscale, University of Science and Technology of China, Hefei 230026, China; Institute of Innovative Materials, Department of Materials Science and Engineering, Department of Chemistry, Southern University of Science and Technology, Shenzhen 518055, China

**Keywords:** ceramic-metal composites, biomimetic materials, nacre-like structures, scalable and shapable properties, tough and strong

## Abstract

Natural nacre that consists of brittle minerals and weak organics exhibits a high fracture toughness while retaining a high strength. The exceptional mechanical performance of nacre is attributed to its hierarchical structure like a ‘brick-and-mortar’ structure, which has inspired the development of tough ceramic-based composites. However, the practical applications of biomimetic structural ceramics are hindered by limited material size, fabrication efficiency and flexibility of being molded into various shapes. We herein report the fabrication of nacre-like ceramic-metal composites based on deformable alumina microspheres coated with nickel salt. Green bodies are produced by assembling the composite microspheres in molds with different shapes. During the hot-pressing sintering of the green bodies, the microspheres are flattened into platelets under pressure and fill up the entire space without visible voids. The aligned platelets are separated by nickel that is reduced from the nickel salt on their surface, constituting a typical ‘brick-and-mortar’ structure. By tuning the microsphere sizes, the microstructures of the composites can be optimized to obtain a high flexural strength (386 MPa at room temperature and 286.86 MPa at 600°C) and a high fracture toughness (12.76 MPa·m^1/2^ at room temperature and 12.99 MPa·m^1/2^ at 600°C) simultaneously. This strategy opens a promising avenue for the feasible mass production and all-in-one molding of nacre-like ceramic-metal composites with various shapes, sizes and raw materials.

## Teaser

Based on the compression of deformable ceramic microspheres into platelets, nacre-like ceramic-metal composites integrating high strength, high toughness and shape adaptability are produced via an all-in-one molding process.

## INTRODUCTION

Ceramics are brittle though renowned for their strength. In contrast, metals are less strong but more ductile [1]. Ceramic-metal composites (cermets), combine the rigidity of ceramics and the ductility of metals, resulting in a material that shows a compromise between strength and toughness [2]. By combining various raw materials, the mechanical performance such as toughness, wear resistance and damping capacity of the cermets can be integrated [3,4]. Nevertheless, because of the conflict between the material strength and its intrinsic toughness, the presence of metals in ceramics leads to strength loss of the cermets. Similarly, ceramics as strengthening additives can make cermets brittle [5,6]. Such conflict has been evidenced in many of the cermets that have been used as engineering materials, which bottlenecks the mechanical performance of the cermets [7]. In contrast, living organisms have developed sophisticated strategies to overcome this conflict [8,9]. For example, mollusk nacre, which consists of brittle aragonite and flexible organics, has a fracture toughness that is 40 times higher than geological aragonite in terms of stress intensity while retaining a high strength [10]. The performance of natural nacre originates from its hierarchical structure that spans several length scales [11,12], such as the brick-and-mortar structure consisting of calcium carbonate platelets and biopolymers, the dovetail structure of the platelets, nano-asperities on the platelets, and mineral bridges between adjacent platelets. Consequently, a combination of toughness and strength has been achieved in natural nacre. The ‘brick-and-mortar’ model derived from nacre has inspired the design and fabrication of various nacre-like composite materials including cermets with not only high strength and toughness but also multiple functions [13].

Despite the improved performance of nacre-like cermets, the practical applications of these materials have been retarded by present fabrication techniques [14]. In nature, mollusks produce nacre via the biomineralization of calcium carbonate in organic matrixes mediated by soluble biopolymers and ions, and it can take several years to precipitate a nacre that is <1 cm thick. To fabricate nacre-like cermets, several technologies have been proposed. The freeze-casting method represents a universal approach to preparing such cermets consisting of different ceramics and metals. The lamellar structure is induced by the growth of ice crystals into layers in a bidirectional temperature gradient, which are then removed via lyophilization. Nacre-like cermets are obtained by infiltrating metals into the lamellar ceramic scaffold [15]. Yet the product size cannot be very large due to the freezing procedure. Coextrusion of nacre-like cermets has been reported as well, but the material is composed of ceramic bars rather than platelets/bricks [16]. The lamellar structure can also be constructed by the controlled assembly of ceramic/metal subunits in external fields, such as magnetic field, gravitational field and electric field [17–19]. However, many of these subunits are rigid micro platelets, which are not possible to form a very dense structure by pressing [20,21]. Besides, the voids left in the structure can cause local stress concentrations, which threaten the mechanical performance of the cermets. A technique that can efficiently fabricate nacre-like cermets with large sizes, dense structures and diverse shapes has not been reported as yet.

**Figure 1.**
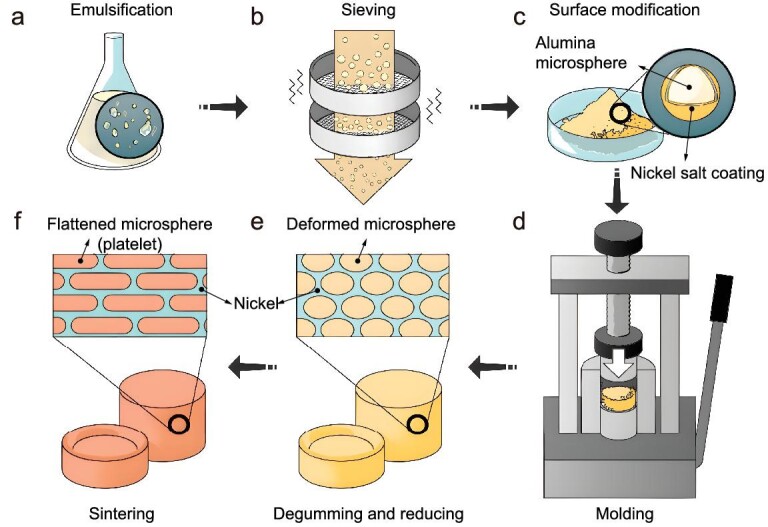
Preparation of the Al_2_O_3_-Ni cermets. (a) Ceramic microspheres stabilized via biopolymer gelation and crosslinking. (b) Microspheres with different sizes are separated by sieving. (c) Microspheres coated with a metal precursor layer. (d) Microsphere powder is shaped in the molds to form green bodies. (e) After degumming with pressureless heat treatment, the metal precursor is also reduced into metallic nickel. (f) Cermets obtained after further sintering, where the microspheres are finally flattened into dense platelets.

Herein, we developed a fabrication strategy for nacre-like cermets based on the assembly of deformable ceramic microspheres coated with metallic salts (Fig. 1, Fig. S1). Rather than the previously reported rigid subunits, these microspheres can be flattened into platelets with fixed pressure and further adapt themselves to adjacent ones to fill up the entire structure under hot-pressing, eliminating the voids between subunits. The prepared cermets exhibit both high strength and high fracture toughness. More specifically, the microspheres are synthesized through the emulsification of ceramic slurry in organic solvents, indicating that the composition and size of the microspheres can be tuned. The microspheres exhibit a porous structure (Fig. S2), which is crucial to their deformation into platelets (Fig. S3). In contrast, the compression of rigid nonporous microspheres always leads to an anisotropic deformation [22]. Besides, the metallic salt coating, which is the precursor of metal in the obtained cermets, can also be feasibly controlled and replaced. These offer an opportunity to control the material structure as well as mechanical behavior more precisely. Furthermore, this strategy allows the all-in-one molding fabrication of large cermets with irregular shapes simply by using more microspheres and irregular-shaped molds (Fig. S1).

## RESULTS AND DISCUSSION

### Nacre-like cermets fabrication via deformable microspheres

Briefly, the bottom-up strategy for cermets fabrication relied on the preparation of ceramic microspheres with proper deformability to transform into platelets, and enough strength to maintain their structural integrity during the compression. The raw material for the microspheres was alumina nanoparticles with an average diameter of ∼50 nm. Under vigorous stirring, the nanoparticle aqueous slurry could spontaneously form microdroplets with the help of surfactants in cyclohexane (Fig. 1a). To solidify these microdroplets, agarose was mixed with nanoparticles in the heated slurry, whereby the microdroplets gelate after cooling. To further stabilize the microdroplets to facilitate the following treatments, sodium alginate was also added to the initial slurry, which was crosslinked in nickel chloride ethanol solution due to the strong interaction between sodium alginate and nickel ions. The obtained microspheres were lyophilized and sieved into different diameters (Fig. 1b, Fig. S4). Then a layer of nickel(II) carbonate hydroxide tetrahydrate was coated on the solidified microspheres (Fig. 1c, Fig. S5), which were subsequently shaped in different molds (Fig. 1d, Fig. S1). The obtained green bodies were then degummed, reduced, and compressed again, whereby the alumina microspheres were slightly flattened (Fig. 1e, Figs S6–S8). Further consolidation was achieved via spark plasma sintering. As the sintering temperature of α-alumina was a little higher than the melting point of nickel metal, the nickel would melt and be squeezed out during the sintering of the composite. To avoid this, a small amount of amorphous silica was added into the initial slurry as the sintering aid, by which the sintering temperature was reduced to 1420°C [23], ∼35°C below the melting point of nickel (Fig. S9). The ceramics were thus densified during the sintering process, and the microspheres were flattened into platelets (Fig. 1f).

### Hierarchical structure of the cermets

**Figure 2.**
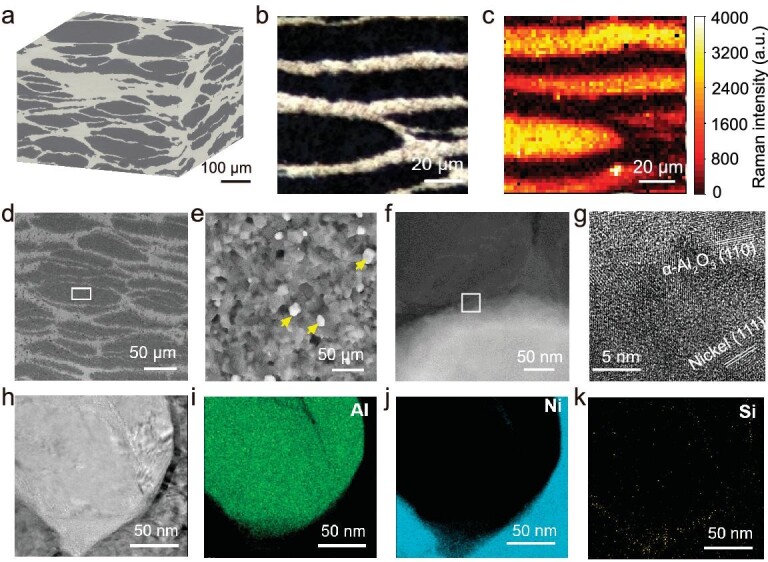
Microstructure analyses. (a) Perspective 3D CT image of a cermet sample, showing the nacre-like structure. The dark areas are alumina, while the bright areas are nickel. (b and c) Cross-sectional structure of the sample revealed by optical microscopy (b) (dark areas: alumina; bright: nickel) and the corresponding Raman mapping (c), where the intensity indicates the α-alumina distribution. These suggest the platelets are α-alumina, which are separated by metallic nickel. (d and e) A polished section of the sample, where the square in (d) is magnified in (e), showing that the platelet consists of ceramic nanoparticles. Yellow arrows indicate the few nickel nanoparticles present in the platelets. (f and g) The ceramic-metal interface structure. A close contact between the two phases is observed in (f); the lattices shown in (g) (square in (f)) suggest they are α-alumina and nickel, respectively. (h–k) Elemental mapping of the ceramic-metal interface. Si is distributed both at the ceramic-metal interface and in the ceramic nanograins.

Energy dispersive spectrometer (EDS) mapping and X-ray diffraction pattern of the cermets suggest the composite consists of α-alumina and metallic nickel (Figs S10 and S11). 3D reconstruction of the cermets revealed a typical nacre-like ‘brick-and-mortar’ structure, where alumina platelets as the bricks were embedded in a continuous nickel mortar (Fig. 2a, Fig. S12). The volume fraction of the ceramic phase was ∼60%. The optical micrograph and the Raman spectral image of the same cross-sectional area of a cermet sample agree with each other, both of which illustrate the nacre-like structure (Fig. 2b and c). As the platelets were transformed from the initial microspheres, the periodicity of the nacre-like structure of the sintered cermets strongly relied on the size of the microspheres. This provides a feasible way to tune the microstructure and the eventual mechanical performance of the nacre-like cermets: the length-to-diameter ratio of the ceramic platelets can switch their failure mode between platelet failure and platelet sliding [24], which in turn influences the strength and fracture toughness of the cermets.

The cermets structure at smaller scales was further observed. Scanning electron microscopy (SEM) images showed the alumina platelets consisted of small crystallites with a relatively narrow size distribution (Fig. S13). A few nickel nanoparticles were dispersed within the ceramic platelets, which were supposed to toughen the platelets via bridging (Fig. 2d and e) [25,26]. The nickel infiltration could be attributed to either the nickel ions incorporated during the preparation of the alumina spheres, or the thermal diffusion during the sintering process. Transmission electron microscopy (TEM) imaging showed a close contact between the ceramic phase and the metallic phase. EDS mapping suggested that Si, which was derived from the sintering aid silica, was not only located inside the alumina nanograins, illustrating the merging of the initial alumina nanoparticles, but also accumulated at the ceramic-metal interface (Fig. 2f–k). High-resolution TEM (HRTEM) imaging of the interface suggested the existence of nickel-aluminum spinel (NiAl_2_O_4_; Fig. S14), a phase that forms via the high-temperature solid-phase interaction between alumina and nickel in the presence of a trace amount of oxygen [27,28]. The elemental composition of this interfacial layer overlapped with both the ceramic phase and the metallic phase, and thus improved the interfacial wettability between the two phases.

### Quasi-static mechanical performance

**Figure 3.**
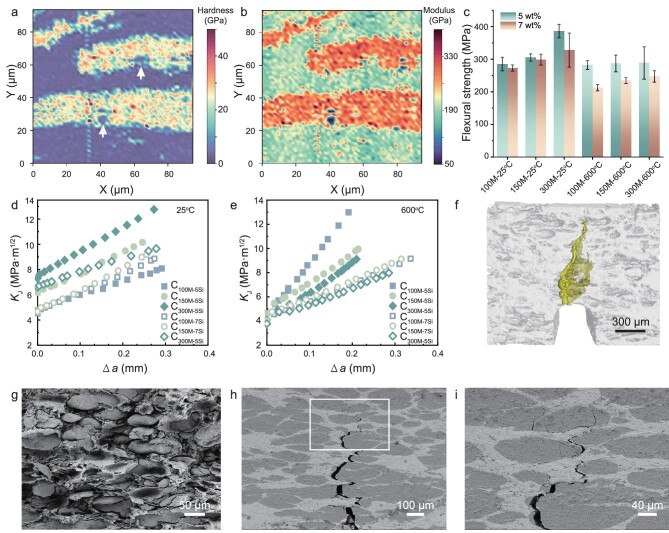
Mechanical properties. (a and b) 2D distribution of hardness (a) and modulus (b) of the sample cross section. White arrows in (a) indicate defects in the ceramic platelets. (c) Flexural strength of the samples with different amounts of sintering aid measured at room temperature and 600°C, respectively. (d and e) Rising crack-extension resistance curves of the cermet samples at (d) room temperature and 600°C (e). (f) Reconstructed CT image showing the crack propagation path. (g) Natural fracture surface of the sample C_300M-5Si_ (room temperature), illustrating the pull-out of the ceramic platelets. (h and i) Crack propagation of the sample C_300M-5Si_ (room temperature), where (i) is the magnified view of the selected area in (h). The propagation path is tortuous because of crack deflection and platelet pull-out can be observed.

Nanoindentation tests of the nacre-like cermets revealed two alternating areas with different hardness and elastic modulus, which corresponded to the rigid alumina platelets and the ductile nickel matrix, respectively (Fig. 3a and b). This structure allowed an extrinsic toughening mechanism of the platelet sliding in the matrix. To spontaneously achieve a high strength and a high toughness, the geometry and the inherent mechanical properties of the two phases as well as their interface should be carefully controlled [29]. To simplify the complicated multiparameter optimization process, we focused on two factors: the geometry of the alumina platelets and the sintering aid contents. To adjust the alumina platelet geometry, three sieves with different mesh sizes (100 M, 150 M, and 300 M; the larger the mesh number, the smaller the mesh size) were used to screen the initial microspheres into three groups (Fig. S4), which were used for the following preparations. The microspheres with smaller size exhibited less deformability and the obtained platelets accordingly had a smaller side-to-thickness ratio (Fig. S15). In contrast, microspheres with larger size could be sufficiently flattened. However, the excessive elongation led to uneven thickness of the platelets, giving rise to potential defects during the platelet failure (Fig. 3a). Notably, despite different platelet geometry, the ceramic contents in the nacre-like cermet samples could be made similar by controlling the ratios of the raw materials. Regarding the content of the sintering aid silica, although it lowered the sintering temperature of the α-alumina, it could also reduce the strength of the sintered alumina platelets [30]. The weight percentages of the silica in the ceramic phase were thus set to 5 wt% and 7 wt%, respectively, as the alumina cannot be sintered properly with a less amount of silica (Fig. S16). Consequently, the obtained cermets were labeled as C_xM-ySi_, where x represented the mesh number, and y represented the silica content.

Due to the diverse applications of cermets such as in the aerospace industry and equipment manufacturing, the working condition of cermets often spans a wide range of temperatures [31–33]. We evaluated the performance of the nacre-like cermets at both room temperature (25°C) and high temperature (600°C). At room temperature, the flexural strength of the C_150M-5Si_ increased by only 7% compared with that of the C_100M-5Si_, while that of the sample C_300M-5Si_ further increased by 26.6%. The flexural strength reached 386 ± 20 MPa (Fig. 3c). The jump in the strength of the C_300M-5Si_ could be ascribed to the proper side-to-thickness ratio that endowed the platelets with high tensile strength. In contrast, the platelet strength of the C_100M-5Si_ and the C_150M-5Si_ were lower due to the defects in the platelets (Fig. 3a). In the presence of excess sintering agent silica, the strength of the alumina platelets decreased [30]. In addition, because Si also piled up at the ceramic-metal interface, the interfacial bonding should be reduced accordingly (Fig. 2k) [34]. The two pathways explained the decreased flexural strength of the samples with 7 wt% silica. At the elevated temperature, the strength of the nickel matrix, the ceramic-metal interface and the alumina platelets were all weakened, resulting in the decreased flexural strength of all samples. Notably, unlike that at room temperature, the flexural strength data of the three samples with 5 wt% silica were similar at 600°C (Fig. 3c, Fig. S17). A possible explanation was that, at the microscale, the fracture of all three samples was dominated by the ceramic-metal interfacial strength, which was reduced by the elevated temperature. Besides, the impact of the temperature on the sample strength was much larger for the samples with excess silica, indicating the Si-containing interfacial phase was temperature sensitive.

The crack resistance of the nacre-like cermets was evaluated by single-edge notched beam bending tests. All the samples showed an extrinsic toughening behavior unraveled by the rising R curves (Fig. 3d and e). Reconstructed computed tomography (CT) imaging of a fractured sample showed the meandering cracking path, indicating more energy was dissipated during crack propagation (Fig. 3f). At room temperature, as the crack extends, the sample C_300M-5Si_ exhibited the highest fracture toughness, 12.76 MPa·m^1/2^. The fractured surface revealed pronounced platelet sliding and large-angle crack deflection; only a few ceramic platelets were penetrated by the crack (Fig. 3g–i). This phenomenon was similar to that observed in natural nacre, where a few aragonite platelets would break after bending tests, indicating an optimization of platelet strength and geometry that led to both high strength and toughness [35]. Indeed, both the flexural strength and the fracture toughness of the C_300M-5Si_ were highest among the samples, highlighting the optimized parameters for the nacre-like cermets at room temperature. The sample C_300M-7Si_ showed lower toughness because of the lower ceramic-metal interfacial strength, whereas the lower toughness of the other four samples could be attributed to platelet failure instead of platelet sliding, which could dissipate a lot of energy, and is the dominating microscopic mechanism of the fracture process. This agreed with the analyses of their flexural strength. Remarkably, while the C_100M-5Si_ had almost the lowest fracture toughness at room temperature, it became the toughest sample at 600°C and the fracture toughness reached 12.99 MPa·m^1/2^. Meanwhile, the toughness of the C_300M-5Si_ decreased notably at the elevated temperature. We proposed that, because the elevated temperature weakened the ceramic-metal interface and softened the nickel matrix, the microscopic fracture mode of the C_100M-5Si_ changed from platelet failure to platelet sliding, and thus more energy was consumed during the sample fracture. In contrast, the platelets in the C_300M-5Si_ could already slide at room temperature, and the weakened interface and the softened matrix at the elevated temperature thus reduced the energy dissipation through platelet sliding. However, due to the lower tensile strength induced by the incorporation of excess silica, the platelets in the C_100M-7Si_ still could not slide properly as evidenced by the limited strain, whereby its fracture toughness was not as high as that of the C_100M-5Si_ with larger strain (Fig. S18).

### Dynamic mechanical performance

**Figure 4.**
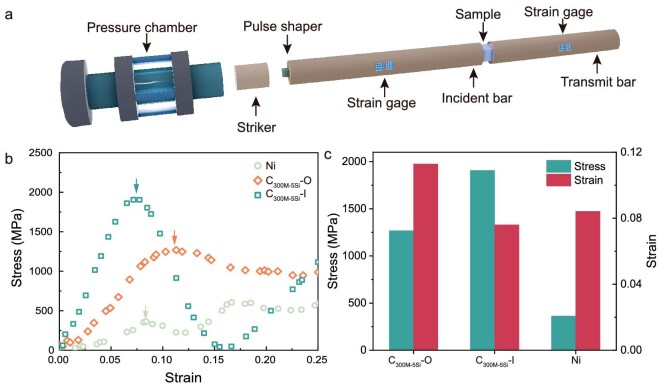
Dynamic mechanical performance. (a) Experimental setup. (b) Stress-strain curves generated by striking the sample perpendicular (C_300M-5Si_-O; orange) and parallel (C_300M-5Si_-I; steel blue) to the alumina platelet respectively, as well as pure nickel. (c) Stress and strain of the samples at the failure point (indicated by the arrows in (b)).

We assessed the dynamic mechanical behavior of the nacre-like cermets under high-strain-rate compression by split-Hopkinson pressure bar tests (Fig. 4a). The C300M-5Si were used for the test because of their high strength and fracture toughness at room temperature. In consideration of the anisotropy of the nacre-like microstructure, the impact experiments were performed in both the in-plane (parallel to the alumina platelets; C300M-5Si-I) and out-of-plane (perpendicular to the alumina platelets; C300M-5Si-O) orientations. In addition, samples made of pure nickel were also used for comparison (Fig. 4b and c). The stress-strain curves indicated distinct dynamic responses along two compression directions (Fig. 4b). Under the out-of-plane impact, the sample was gradually compressed without catastrophic damage, indicating that the slipping of the alumina platelets and the plastic deformation of the nickel matrix could absorb the impact energy stably and effectively. When the strain was larger than 0.15, the stress-strain curve suggested the sample underwent a long-range plastic flow deformation before the final densification (Fig. S19a). Under the in-plane compression, however, the sample was crushed within a short range, which was evidenced by the stress-stain curve of the C300M-5Si-I that the stress fell to almost zero at the strain of 0.16 (Fig. 4b). The higher impact stress of the in-plane compression originated from the strong ceramic tablets parallel to the impact direction (Fig. 4c). In contrast, the impact stress of pure nickel was much lower than that of the cermets because of the absence of alumina. After being crushed, the sample could be further compressed and the stress-strain curve rose again as the nickel matrix could maintain the integrity of the sample, though the alumina platelets were crushed (Fig. S19b and c).

## CONCLUSION

**Figure 5.**
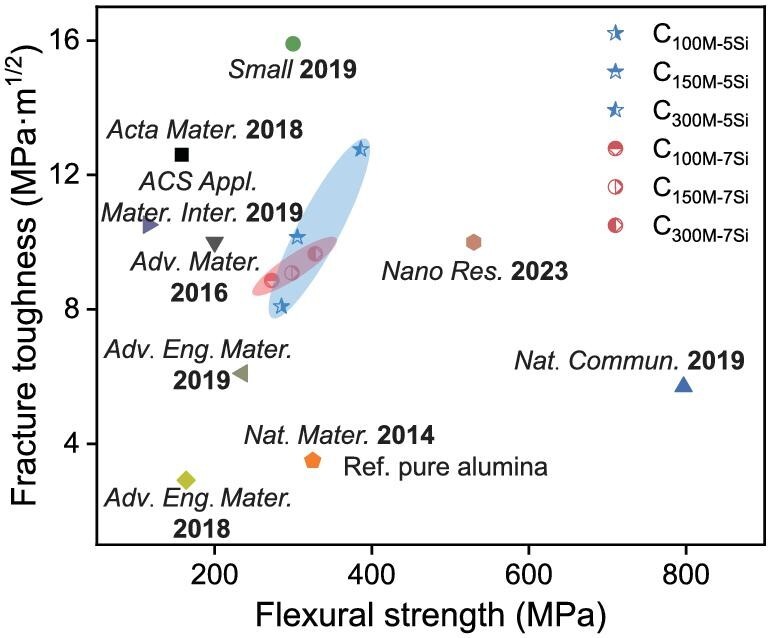
Ashby plot. A comparison of strength and fracture toughness between the Al_2_O_3_-Ni cermet in this work and previously reported cermets [16,18,41–45]. The current Al_2_O_3_-Ni cermet exhibits both high strength and fracture toughness, and is among the best cermets.

Although the nacre-like cermets produced via the compression of deformable microspheres do not show the highest strength and fracture toughness, their mechanical performance is already among the best ceramic-based composites (Fig. 5). Importantly, while the applications of many biomimetic structural ceramics are restrained by the material size, this strategy has a potential to fabricate large nacre-like cermets via using more microspheres. By combining with additive manufacturing techniques such as 3D printing, this strategy stands a chance of creating cermets with not only local surface curvature but also local mechanical anisotropy, which has not been possible through many previous fabrication techniques [36–38]. In addition, as this work has shown that temperature-dependent optimization can be achieved by tuning the microsphere size and the sintering aid content, nacre-like cermets with structural gradients that meet the challenges of temperature gradients inside the materials caused by extreme service environments can be produced [39]. Besides ceramic-metal composites, different raw materials can be applied to make various nacre-like composites such as pure ceramic composites for diverse applications [40]. We thus anticipate that this feasible and extendable strategy paves the way for the practical applications of nacre-like ceramics.

## METHODS

### Materials preparations

Alumina nanoparticles (∼50 nm in diameter) were bought from Anhui Zhonghang Nanotechnology Development Co., Ltd. Sodium alginate, agarose, ammonium bicarbonate and cyclohexane were purchased from China National Pharmaceutical Group Co., Ltd. Silica (40 wt% aqueous solution) was purchased from Millipore Sigma of Merck Ltd. Nickel chloride and span-80 were purchased from Shanghai Aladdin Biochemical Technology Co., Ltd. All materials were used without further purification.

### Characterization

SEM images were taken with a ZEISS GeminiSEM 450 scanning electron microscope. EDS data were collected using a ZEISS GeminiSEM 450 variable pressure SEM outfitted with an Oxford ULTIM MAX100 X-ray energy spectrometer. TEM samples were prepared by a Helios G4 UX Focused Ion Beam instrument produced by Thermo Fisher Scientific. TEM images were taken with a Thermo Fisher Scientific Talos F200X transmission electron microscope. The Renishaw Raman system was used to obtain the Raman spectra and mappings. The Raman mapping was acquired by a 100× objective using a 532 nm laser with a spot size of ∼300 nm. WiRE 5.5 software was used for data analysis, creating a histogram of the integrated intensity, and achieving the Raman mapping. The Xradia 520 Versa was used to obtain X-ray micro-computed tomography (micro-CT) images. Dragonfly software was used for 3D reconstruction. The cermets densities were evaluated by the Archimedes method.

### Mechanical tests

The three-point bending tests were performed on an Instron 5565 A system equipped with a 500 N load cell. The nacre-like cermets were cut into bar-shaped specimens (∼2 mm × 2 mm × 20 mm) using a diamond cutting saw produced by Shengyang Kejing Auto-instrument Co. For single-edge notched bending tests, the specimens were notched using a wire cutter and sharpened by a razor blade. The depth of the groove is ∼1 mm, and the width is ∼0.3 mm. The loading rate was 0.1 mm·min^−^^1^ for unnotched specimens, and 0.01 mm·min^−^^1^ for notched specimens. The span of three-point bending tests was 12 mm. Each sample was tested at least three times. The microscopic mechanical properties of the nacre-like cermets were measured by nanoindentation tests using a Bruker TI 980 TriboIndenter. The hardness and modulus distribution maps of the samples were obtained with a dot pitch of 1.5 μm and a maximum load of 2 mN. The dynamic mechanical performance of the composites was assessed by an SHPB device under the impact speed of 20.5 m/s. The samples were cut into blocks (4 mm × 2 mm × 2 mm), and each sample was tested at least three times.

## Supplementary Material

nwaf006_Supplemental_File
