# Supplementary material for: Scalable and shapable nacre-like ceramic-metal composites based on deformable microspheres
Source: Natl Sci Rev. 2025 Jan 20;12(3):nwaf006. doi: 10.1093/nsr/nwaf006 (PMC11837330; doi:10.1093/nsr/nwaf006)
Supplement: nwaf006_Supplemental_File [file nwaf006_supplemental_file.pdf]

Supplementary Information

Scalable and shapable nacre-like ceramic-metal composites based on deformable microspheres

Yu-Jie Lu[1†], Xiang-Sen Meng[1†], Qiu-An Sun[3], Jie Wang[1], Jun-Jie Song[3], Peng-Fei Wang[4], Guo-Rui Wang[4,5], Cheng-Xin Yu[1], Yong-Sheng Zhang[3], Li-Bo Mao[1*], Shu-Hong Yu[1,2*]

[1]Department of Chemistry, New Cornerstone Science Laboratory, Institute of Biomimetic Materials & Chemistry, Anhui Engineering Laboratory of Biomimetic Materials, Division of Nanomaterials & Chemistry, Hefei National Research Center for Physical Sciences at the Microscale, University of Science and Technology of China, Hefei 230026, China.

[2]Institute of Innovative Materials, Department of Materials Science and Engineering, Department of Chemistry, Southern University of Science and Technology, Shenzhen 518055, China.

[3]State Key Laboratory of Solid Lubrication, Lanzhou Institute of Chemical Chinese Academy of Sciences, Lanzhou, 730000, China.

[4]Department of Modern Mechanics, CAS Key Laboratory of Mechanical Behavior and Design of Materials, University of Science and Technology of China, Hefei, 230026, China

[5]State Key Laboratory of Nonlinear Mechanics, Institute of Mechanics, Chinese Academy of Science, 15 Beisihuan West Road, Beijing 100190, China.

***Corresponding authors**. E-mails: maolb@ustc.edu.cn; shyu@ustc.edu.cn or yush@sustech.edu.cn

†Equally contributed to this work.

Fabrication of Alumina microspheres

Ceramic slurry was prepared by mixing alumina nanoparticles, silica, sodium alginate and de-ionized water. For the preparation of the $C_{100M-5Si}$, $C_{150M-5Si}$ and $C_{300M-5Si}$, the amounts of the four components are 28.5 g, 1.5 g, 3 g and 100 mL respectively; for the preparation of the C100M-7Si, C150M-7Si and $C_{300M-7Si}$, the amounts are 27.9 g, 5.25 g, 3 g and 100 mL respectively. The mixture was ball-milled for 12 h to ensure the components were sufficiently dispersed to form slurry. This slurry was then mixed with equal volume of 2 wt.% agarose aqueous solution at 65 ºC, and then stirred vigorously until fully mixed. 7 mL of span-80 and 100 mL of cyclohexane were added into the system, which was then incubated for 10 minutes and stirred for another 15 minutes to obtain the water-in-oil emulsion. The emulsion was dripped into nickel chloride ethanol solution (10 g in 500 mL). In this process, the sodium alginate was crosslinked by the nickel ions, and the agarose solidified as it turned cold, so that stable microspheres could be obtained.

Surface modification of the microspheres

4 g of vacuum-dried microspheres were dispersed into 600 mL of 0.33 M $NiCl_2$ aqueous solution, and then 1.2 M NH_4HCO_3 was dropped into the mixed solution until no new bubble was generated. The following reaction occurred in this process:

$$3NiCl_2 + 6NH_4HCO_3 + H_2O = NiCO_3{\cdot}2Ni(OH)_2{\cdot}2H_2O + 6NH_4Cl + 5CO_2\uparrow$$

After the reaction was finished, the precipitant was collected by suction filtration, cooled in liquid nitrogen, and then placed in a Scientz-18N freeze dryer for three days to obtain thoroughly dried microspheres with a layer of nickel(II) carbonate hydroxide tetrahydrate on the surface.

Shaping and degumming

The surface-modified microspheres were placed in a mold with desired size and shape, hot-pressed at 60 ºC for 12 hours at a pressure of 10 MPa to produce the green body. Then the green body was placed in a miniature tube furnace (Anhui CHEMN Instrument Co., Ltd.) and heated to 600 °C for three hours with fresh air blowing into the tube to remove the organics, followed by three hours of hydrogen/argon mixture

flushing (5 vol.% of hydrogen) at 700 °C to reduce the nickel oxides into metallic nickel. After degumming, the green body became loose and fragile, and thus further hot-pressing was applied to maintain its integrity.

Spark plasma sintering

The spark plasma sintering process was performed on an SPS-20T-10-IV sintering furnace produced by Shanghai Chenhua Technology Corp., Ltd. The green body was placed in a graphite die, and alumina powder was filled around the sample. The temperature of the heated sample is measured by an infrared pyrometer through the measuring hole in shell of the mold. To keep metallic nickel from being oxidized at a high temperature, a vacuum purging at 10^{-3} MPa was executed before the sintering process. Nitrogen was used as a protective atmosphere throughout the process. After cooling, the nacre-like cermets were obtained.

Calculation of the fracture toughness K_{Jc} and the crack extension resistance curve

Based on the load-displacement curves obtained from the SENB test, the crack extension resistance curve (R-curve) of the material can be calculated, which can visualize the toughening effect of the material [1]. The J-integral-based calculation of the R-curve is carried out as follows [2]. First, the plane strain fracture toughness K_{Ic} at the onset of crack initiation is calculated by

$$K_{\mathrm{Ic}} = \frac{PS}{BW^{\frac{3}{2}}} f\left(\frac{a}{W}\right),$$

where P is the applied load at the crack initiation point, S the span, B the width of the SENB specimen, W the thickness, and a the initial crack length. For SENB specimens, the initial crack length a is equal to the notch depth.

The function f in the above equation is given by

$$f\left(\frac{a}{W}\right) = \frac{3\left(\frac{a}{W}\right)^{\frac{1}{2}}\left[1.99-\frac{a}{W}\left(1-\frac{a}{W}\right)\left(2.15-3.93\frac{a}{W}+2.7\left(\frac{a}{W}\right)^{2}\right)\right]}{2\left(1+2\frac{a}{W}\right)\left(1-\frac{a}{W}\right)^{\frac{3}{2}}}.$$

The elastic component of J-integral, J_{el}, is defined by the following equation:

$$J_{\mathrm{el}} = \frac{K_{\mathrm{Ic}}^{2}}{E'}.$$

In the above equation, the reduced elastic modulus E' is given by

$$E' = \frac{E}{1-v^2},$$

where E is the elastic modulus and v the Poisson's ratio.

The plastic component J_{pl} at specific displacement point is defined by

$$J_{\mathrm{pl}} = \frac{2A_{\mathrm{pl}}}{Bb},$$

where A_{pl} is the area of plastic region under the load-displacement curve, and b the remaining thickness of the ligament.

The converted stress intensity factor K_{Jc} at these selected points is obtained by

$$K_{\mathrm{Jc}} = \sqrt{(J_{el} + J_{pl})E'}.$$

The corresponding crack length at the above points is calculated using a recursive relationship

$$a_n = a_{n-1} + \frac{W-a_{n-1}}{2}\frac{C_n-C_{n-1}}{C_n}.$$

The compliance Cn is given by

$$C = \frac{u}{f},$$

where u is the displacement and f the load at each point.

The R-curve is obtained by taking the crack extension length a as the x-axis coordinate, and the converted stress intensity factor K_{Jc} as the y-axis coordinate[2].

Figures

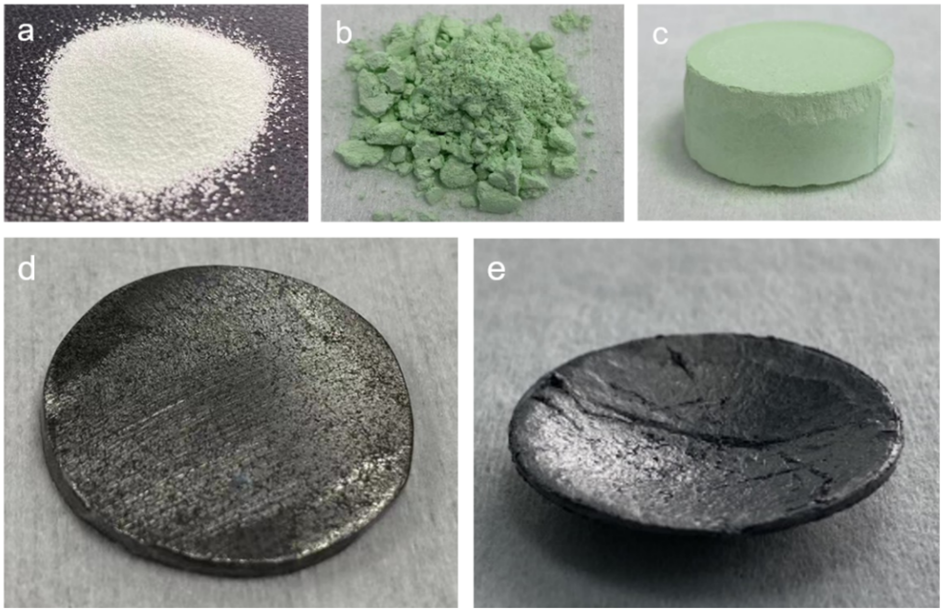

Figure S1. Sample preparation at different stages. (a) Alumina microspheres. (b) Alumina microspheres with metallic salt coating. (c) Green body prepared by hot pressing. (d-e) Images of d disc-shaped, and e bowl-shaped samples produced by different molds.

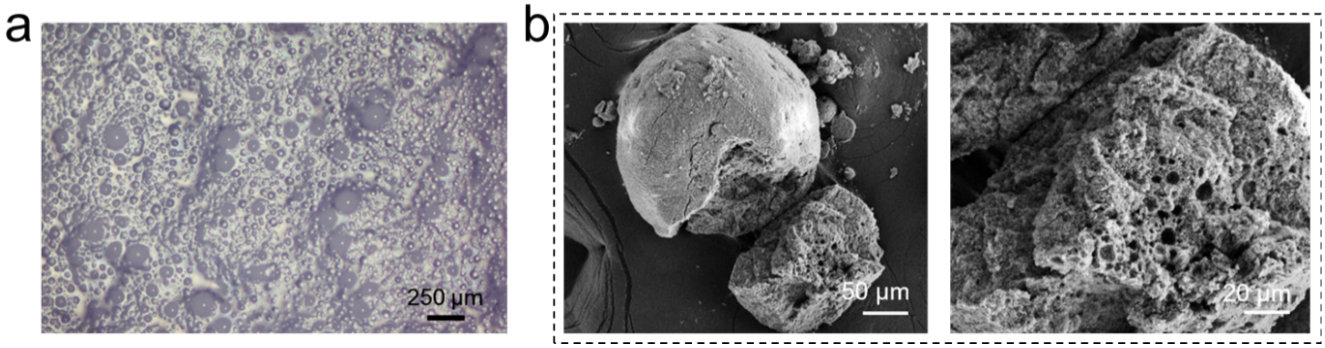

Figure S2. Alumina microspheres. (a) Alumina microspheres. (b) Broken alumina microspheres revealing the porous structure.

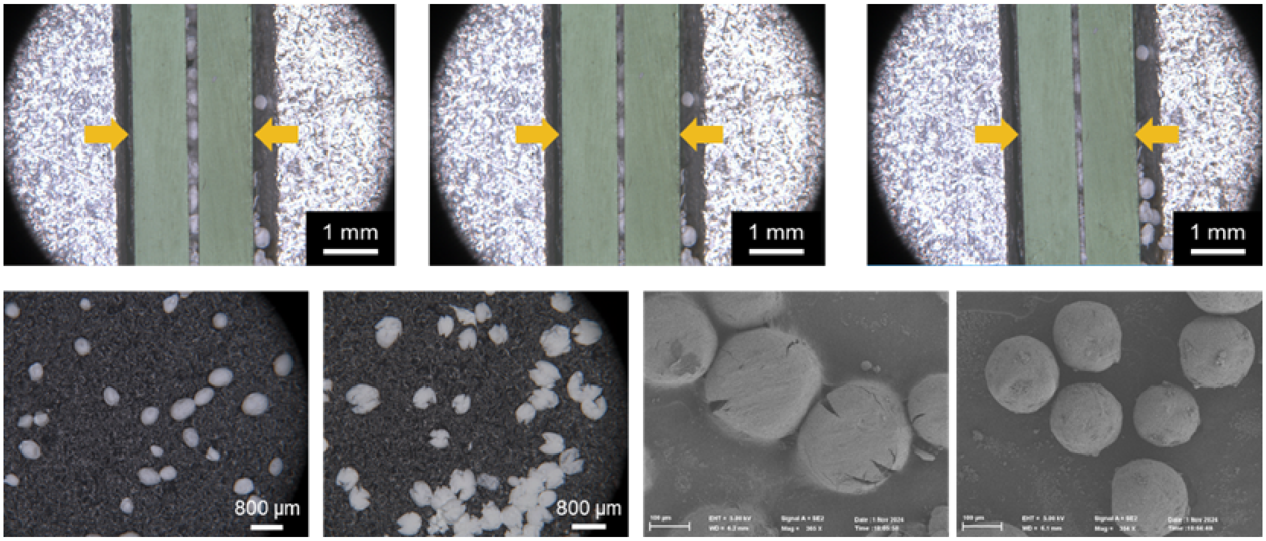

Figure S3. Deformation process of the alumina microspheres. The spheres can be easily flattened by pressing with two glass plates.

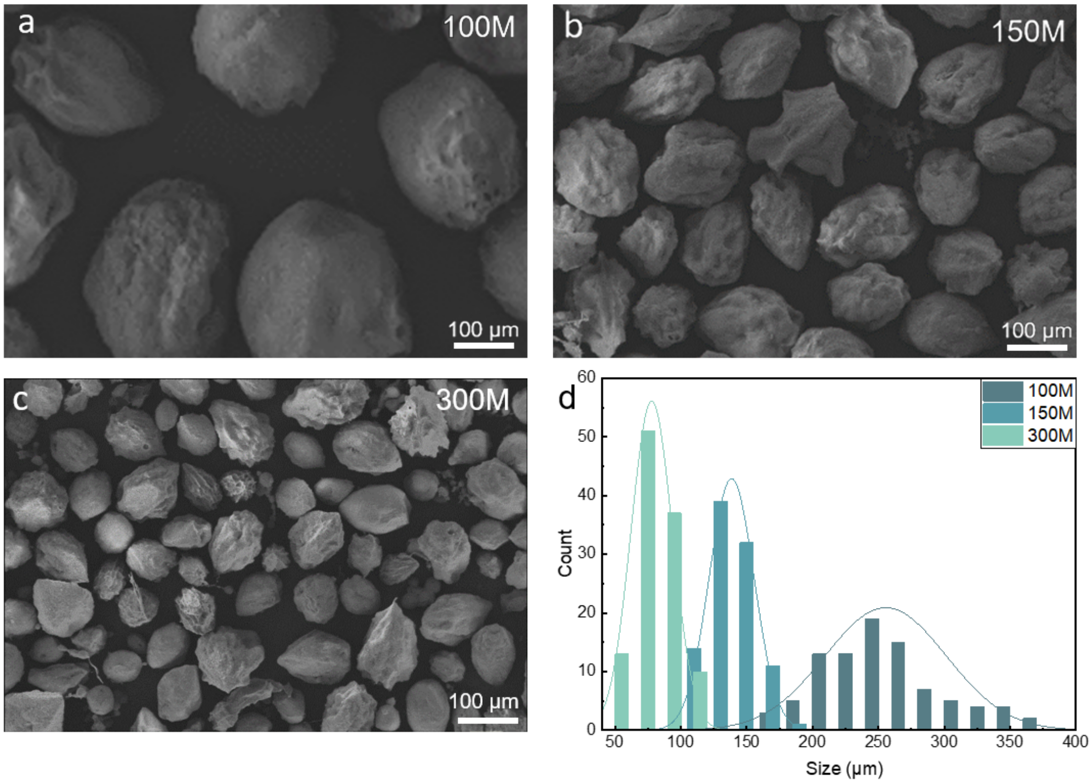

Figure S4. Alumina microspheres with different particle sizes and their particle size distributions. (a-c) SEM images of alumina microspheres separated with 100 mesh (a), 150 mesh (b), and 300 mesh (c). (d) Histogram of particle size distribution of different samples.

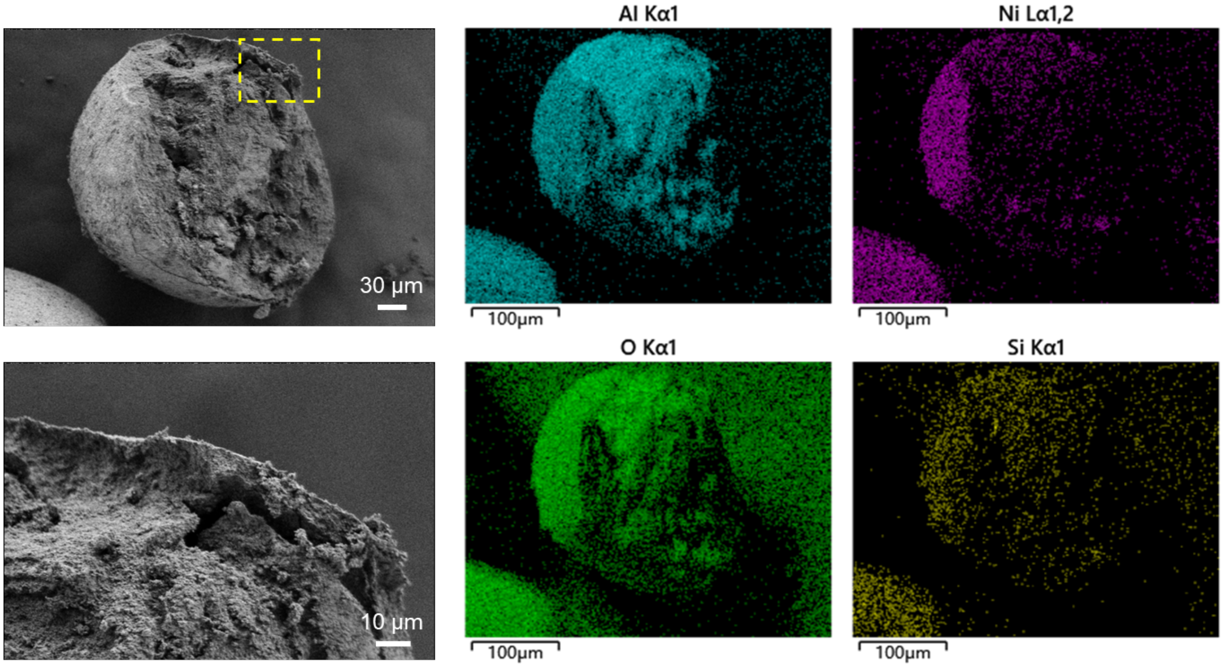

Figure S5. Alumina microspheres with nickel (II) carbonate hydroxide tetrahydrate coating.

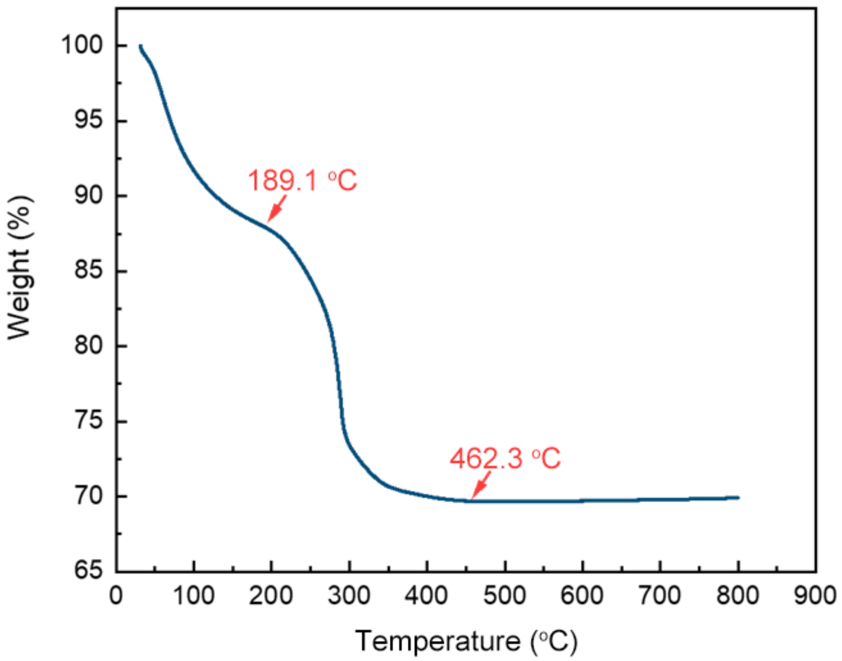

Figure S6. TGA data of the Al_2O_3 microspheres coated with nickel (II) carbonate hydroxide tetrahydrate. The organic components in the sample are oxidized as the temperature increases until the weight of the sample stabilizes at 462.3 ℃.

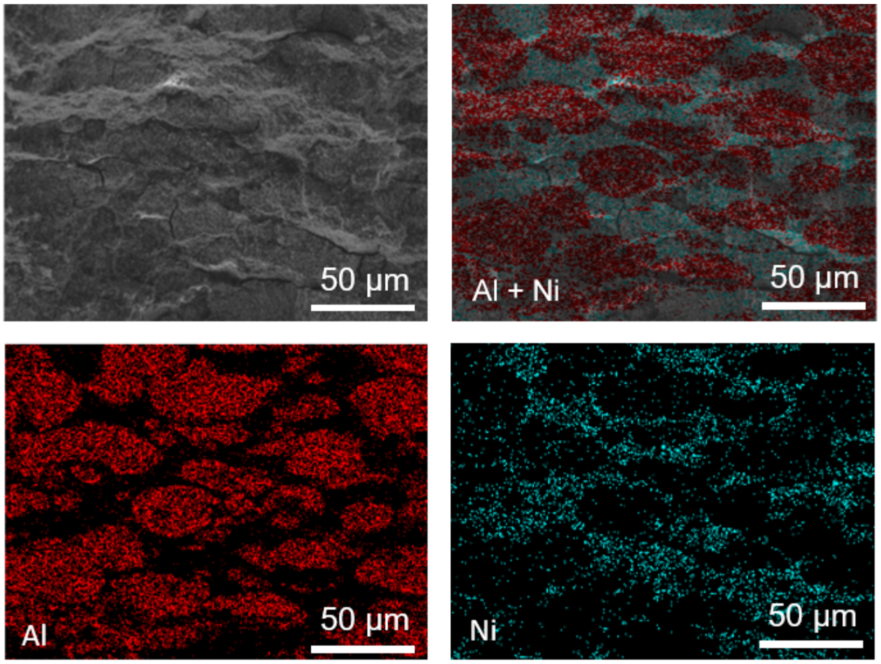

Figure S7. Degummed greed body. SEM image of a cross section and EDS images of the cross section, depicting the distribution of aluminum (Al) and nickel (Ni).

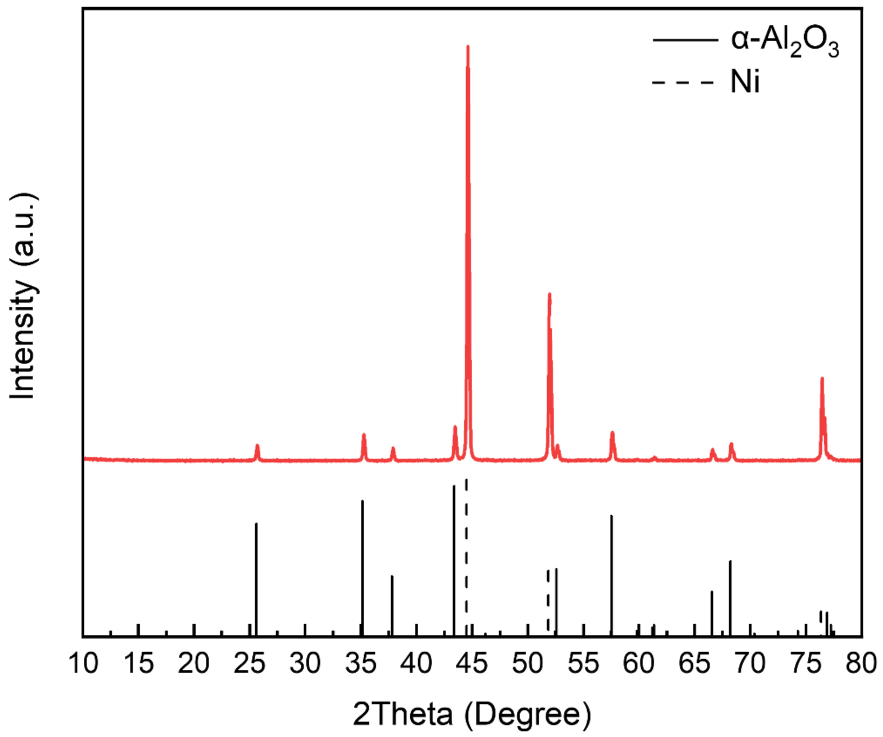

Figure S8. XRD pattern of the green body. Note that nickel(II) carbonate hydroxide tetrahydrate has been reduced to metallic Ni.

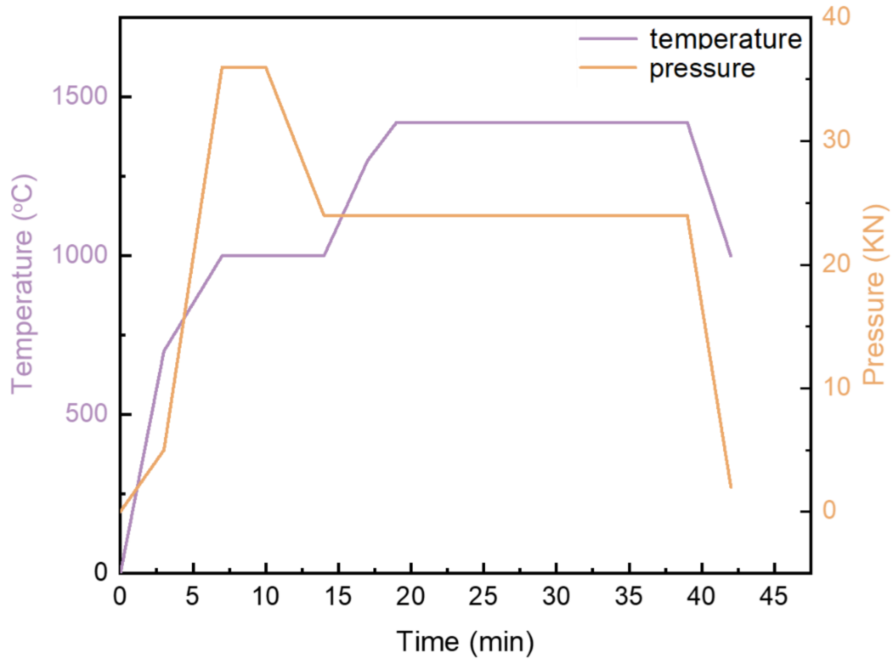

Figure S9. Sintering process of the samples, showing the temperature and pressure control curve over time.

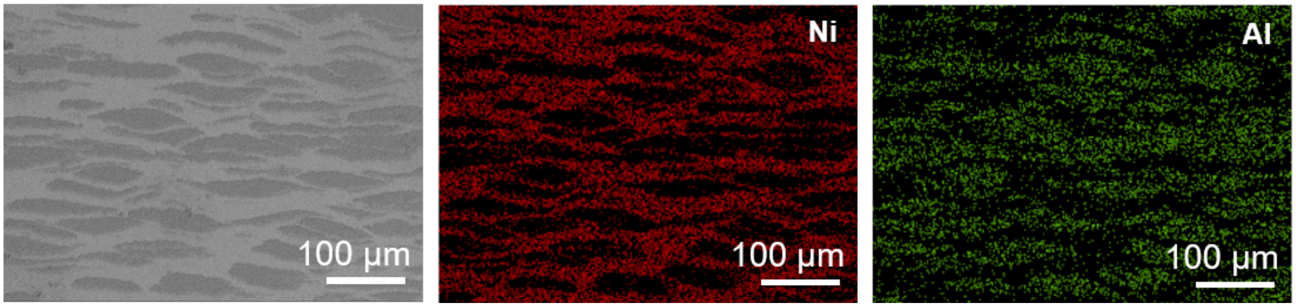

Figure S10. Polished cross-section of the cermets. After sintering, alumina microspheres are transformed into platelets and aligned with each other.

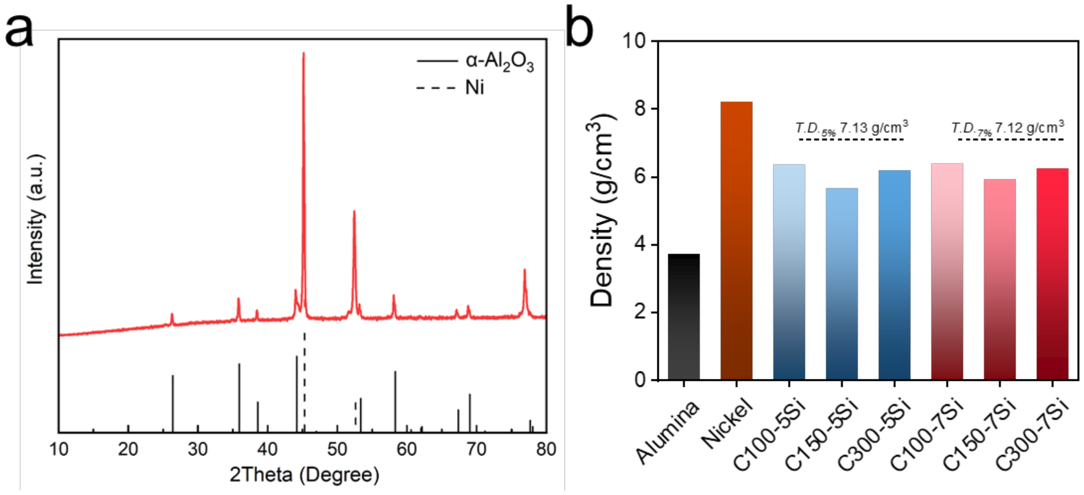

Figure S11. (a) XRD pattern of the cermet $C_{100M\text{-}5Si}$. (b) Density of the cermets and comparison sample. *T.D.* was the abbreviation for theoretical density.

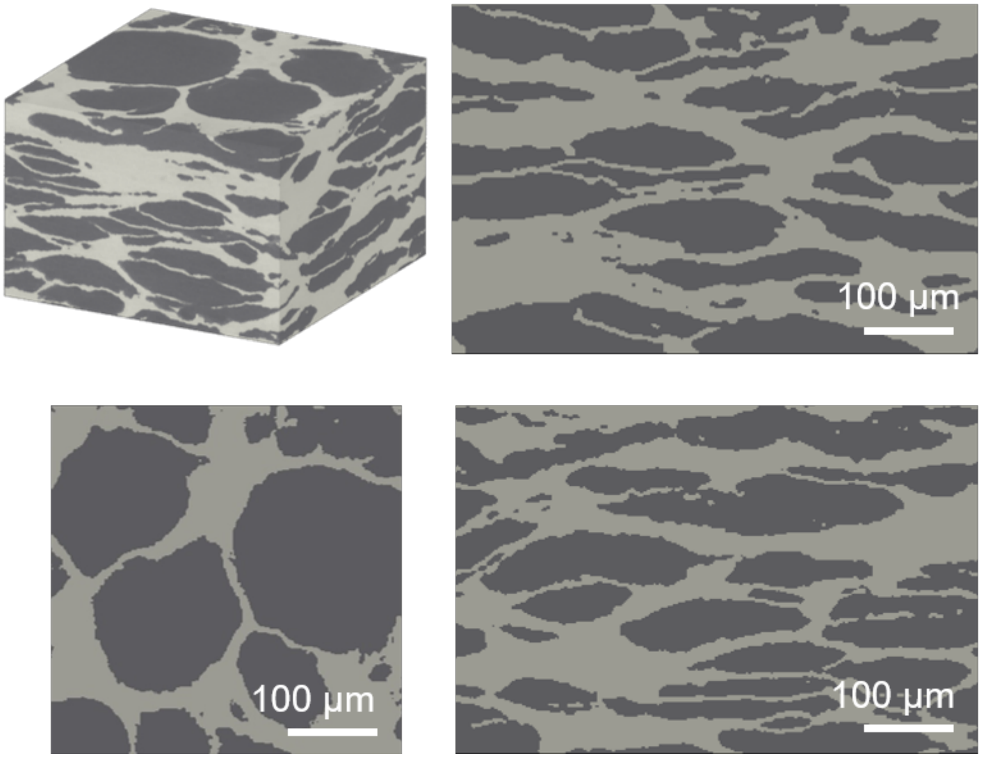

Figure S12. 3D perspective X-ray tomographic images of the cermets.

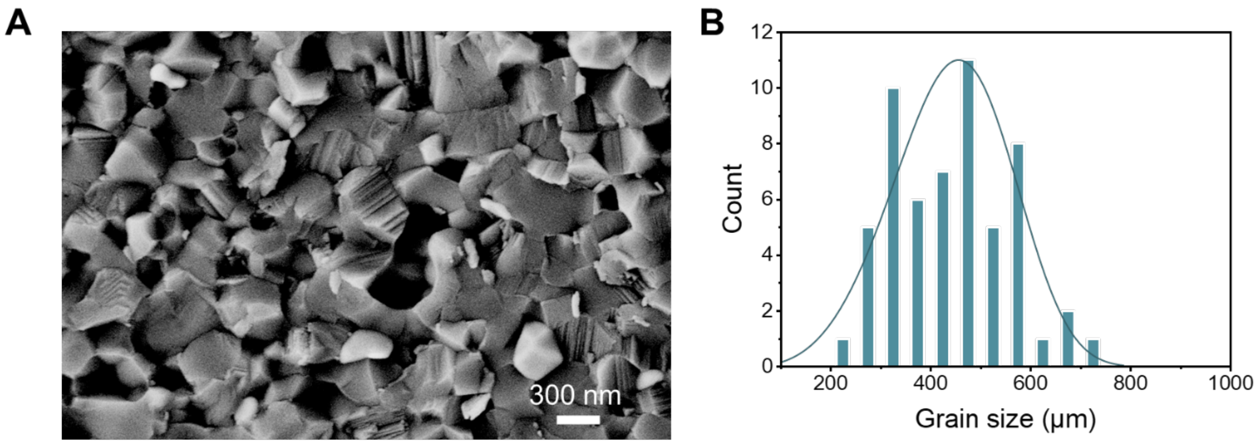

Figure S13. Alumina particle size distribution of the cermets.

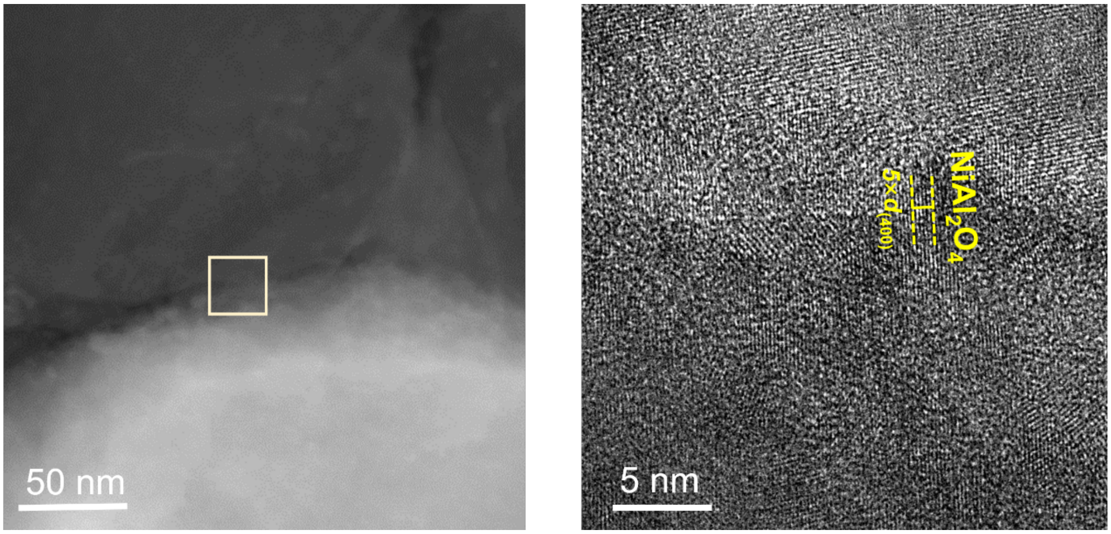

Figure S14. HRTEM images of the ceramic-metal interface showing the possible existence of $NiAl_2O_4$. The formation of $NiAl_2O_4$ follows this equation: $Al_2O_3 + Ni + 1/2\ O_2 = NiAl_2O_4$ [3].

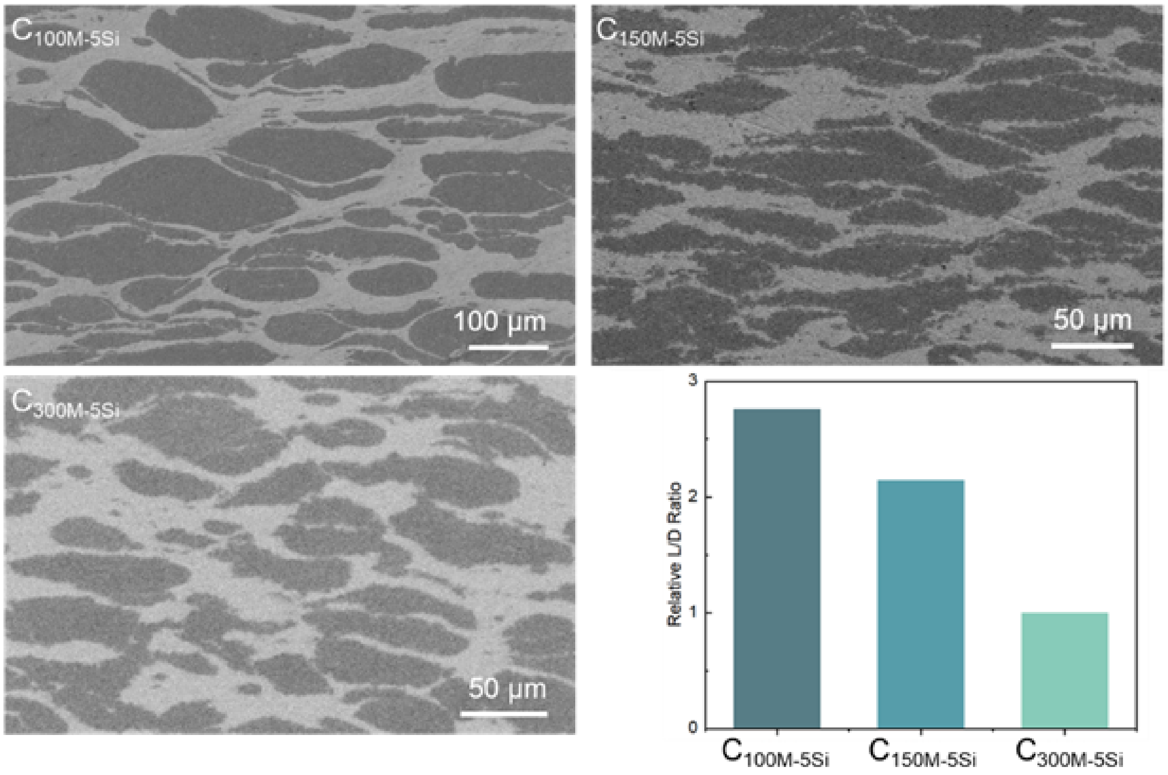

Figure S15. Side-to-thickness ratio of the alumina platelets in different cermet samples.

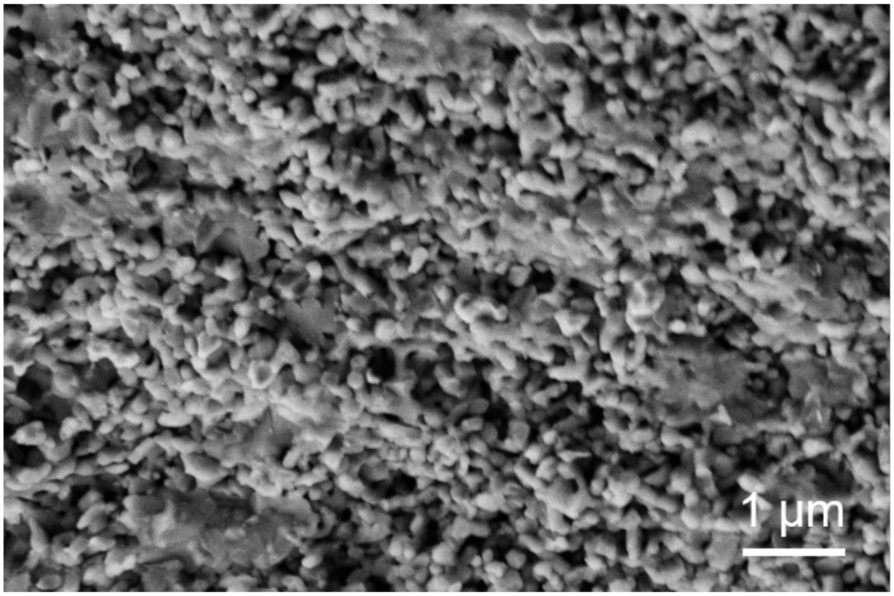

Figure S16. In the presence of 2 wt.% sintering agent silica, alumina nanoparticles cannot be sintered properly.

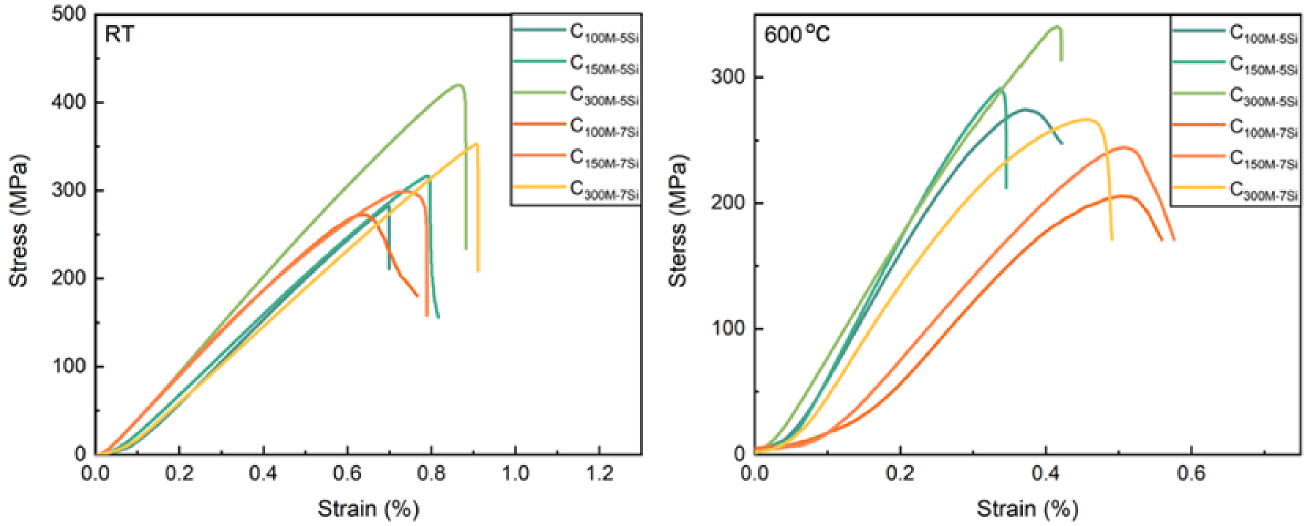

Figure S17. Three-point bending test. (a-b) Stress-strain curves of the Al_2O_3-Ni cermets at room temperature (a) and 600 °C (b).

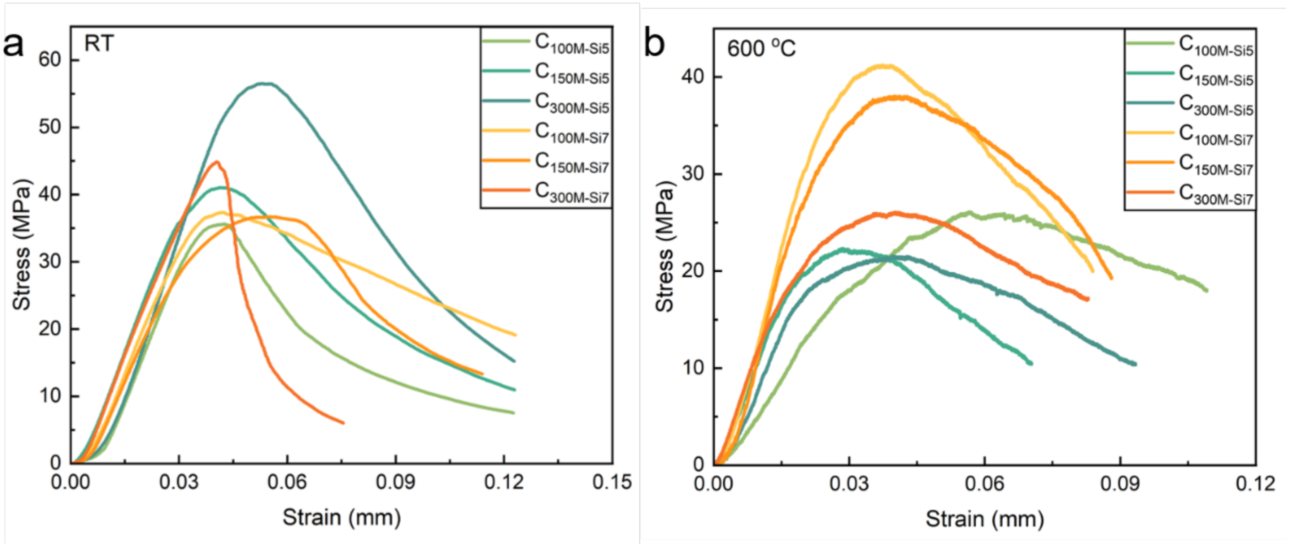

Figure S18. SENB test. (a-b) Stress-strain curves of the Al_2O_3-Ni cermets at room temperature (a) and 600 ℃ (b).

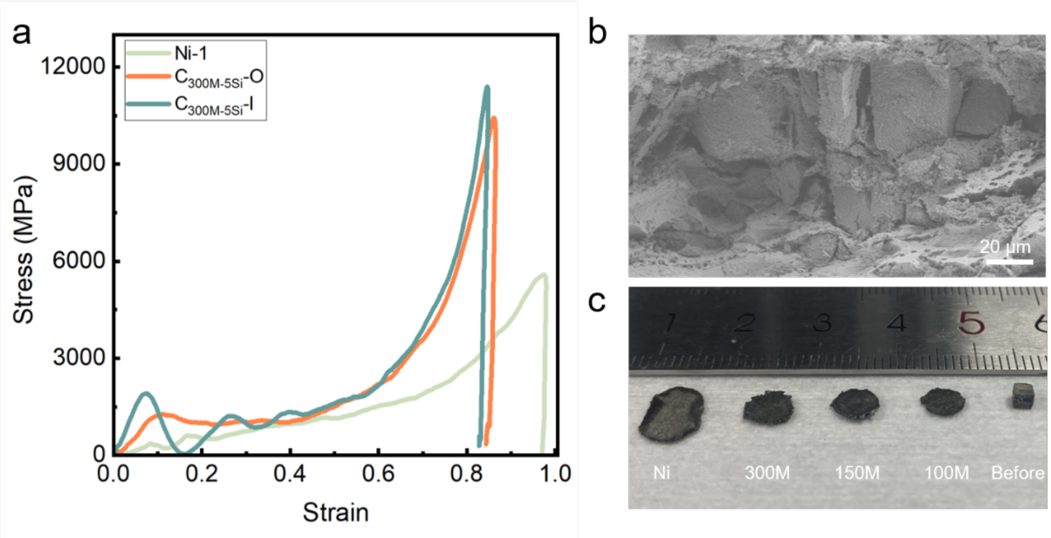

Figure S19. Dynamic mechanical performances of the Al_2O_3-Ni cermets. (a) Stress-strain curves generated by striking the sample perpendicular ($C_{300M\text{-}5Si}$-O) and parallel ($C_{300M\text{-}5Si}$-I) to the ceramic platelet direction. (b) SEM image of the sample after the test. (c) Comparison of the appearance of the samples before and after the test.

References

1. Ritchie RO. The conflicts between strength and toughness. *Nat. Mater*. 2011; **10**: 817-822.
2. Bouville F, Maire E, Meille S *et al.* Strong, tough and stiff bioinspired ceramics from brittle constituents. *Nat. Mater*. 2014; **13**: 508-514.
3. Lieberthal M, Kaplan W D. Processing and properties of Al_2O_3 nanocomposites reinforced with sub-micron Ni and $NiAl_2O_4$. *Mater. Sci. Eng. A* 2001; **302**: 83-91.
